# Elevated NT-proBNP predicts unfavorable outcomes in patients with acute ischemic stroke after thrombolytic therapy

**DOI:** 10.1186/s12883-023-03222-6

**Published:** 2023-05-23

**Authors:** Zhuang Zhu, Bilal Muhammad, Bo Du, Ning Gu, Tian-Yue Meng, Shu Kan, Ying-Feng Mu, Yan-Bo Cheng, Shi-Guang Zhu, De-Qin Geng

**Affiliations:** 1 School of Clinical Medicine, Xuzhou Medical University, Xuzhou, 221000 China; 2 Department of Neurology, Affiliated Hospital of Xuzhou Medical University, Xuzhou, 221000 China

**Keywords:** NT-proBNP, Early neurological deterioration, Prognosis, Acute ischemic stroke, Intravenous thrombolysis

## Abstract

**Objective:**

Few studies correlated n-terminal pro-brain natriuretic peptide (NT-proBNP) with early neurological deterioration (END) and prognosis of acute ischaemic stroke (AIS) patients with rt-PA intravenous thrombolysis. Therefore this study aimed to investigate the relationship between NT-proBNP and END, and prognosis after intravenous thrombolysis in patients with AIS.

**Methods:**

A total of 325 patients with AIS were enrolled. We performed the natural logarithm transformation on the NT-proBNP [ln(NT-proBNP)]. Univariate and multivariate logistic regression analyses were performed to assess the relationship between ln(NT-proBNP) and END, and prognosis and receiver operating characteristic (ROC) curves were used to show the sensitivity and specificity of NT-proBNP.

**Results:**

After thrombolysis, among 325 patients with AIS, 43 patients (13.2%) developed END. In addition, three months follow-up showed a poor prognosis in 98 cases (30.2%) and a good prognosis in 227 cases (69.8%). Multivariate logistic regression analysis showed that ln(NT-proBNP) was an independent risk factor for END (*OR* = 1.450,95%CI:1.072 ~ 1.963, *P* = 0.016) and poor prognosis at three months follow-up (*OR* = 1.767, 95%*CI*: 1.347 ~ 2.317, *P* < 0.001) respectively. According to ROC curve analysis, ln(NT-proBNP) (AUC 0.735, 95%*CI*: 0.674 ~0.796, *P* < 0.001) had a good predictive value for poor prognosis, with a predictive value of 5.12 and sensitivity and specificity of 79.59% and 60.35% respectively. When combined with NIHSS to predict END(AUC 0.718, 95%*CI*: 0.631 ~ 0.805, *P* < 0.001) and poor prognosis(AUC 0.780, 95%*CI*: 0.724 ~ 0.836, *P* < 0.001), the predictive value of the model is further improved.

**Conclusion:**

NT-proBNP is independently associated with END and poor prognosis in patients with AIS following intravenous thrombolysis and has a particular predictive value for END and poor prognosis.

## Introduction

Stroke is the second leading cause of disability and death worldwide, with a recent study showing that in 2020 it is estimated that 3.4 million stroke events, 17.8 million stroke patients, and 3.4 million stroke deaths will occur in people over 40 years of age in China [1, 2]. Intravenous thrombolysis, such as recombinant tissue plasminogen activator (rt-PA) within 4.5 h is an effective treatment method for AIS [3]. However, it has been reported [4–6] that within 24–72 h after intravenous thrombolysis, 3.0%-32.8% of AIS patients experienced early neurological deterioration (END). Additionally, research has demonstrated a clear correlation between END and a higher risk of functional disability, death, and a worse clinical result in AIS after three months [5, 7, 8]. Current approaches to identifying high-risk individuals in AIS patients rely heavily on assessing clinical features and neuroimaging present at onset, the patient's initial response to treatment, and relative biomarkers. Therefore, identifying new biomarkers to forecast AIS patients' prognoses is crucial for clinical treatment planning and intervention.

NT-proBNP is an inert fragment broken down by brain natriuretic peptide (BNP) and then released from the stretched ventricular myocardium. Because of its longer half-life, higher plasma concentration, and superior diagnostic resolution, NT-proBNP is the preferable biomarker over BNP. Stretch stimulation, such as volume overload and hemodynamic stress, raises NT-proBNP levels [9]. The prognosis of patients with cardioembolic stroke and AIS is correlated with NT-proBNP, according to earlier studies [5, 10]. However, little is known about the predictive ability of NT-proBNP in AIS treated with intravenous thrombolysis. Therefore, this study aimed to investigate NT-proBNP levels' predictive power in AIS patients following intravenous thrombolysis.

## Methods

### Subjects

**Fig.1:**
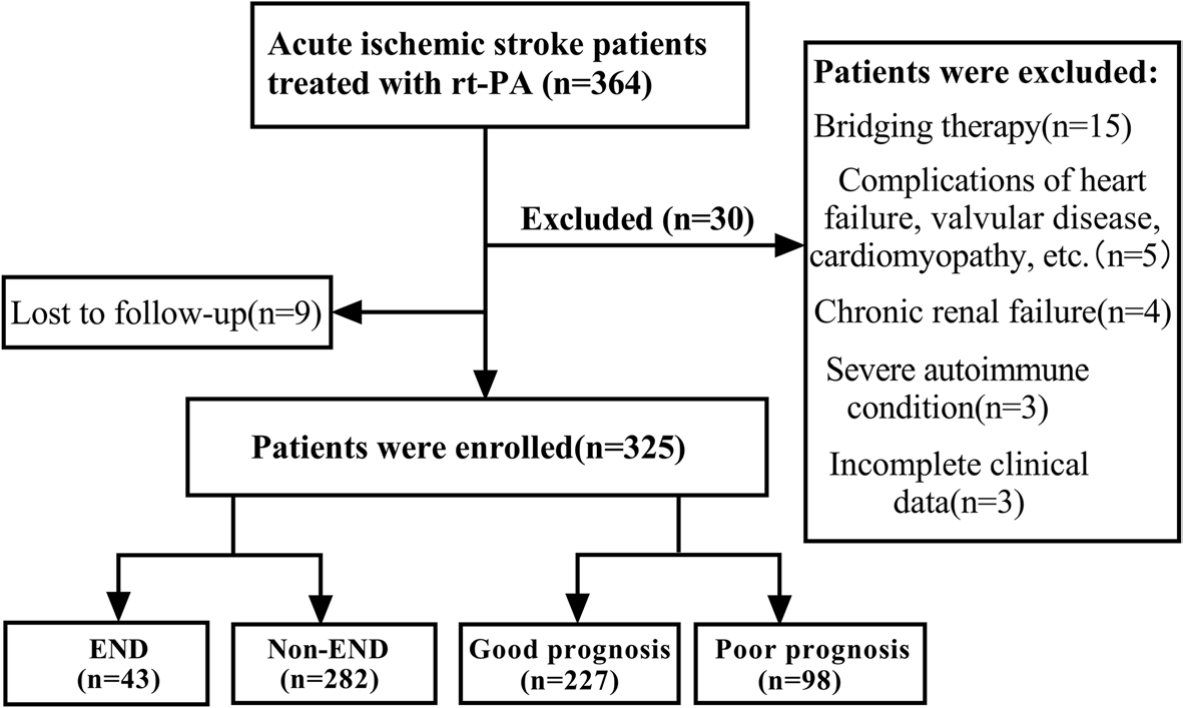
Flow chart for retrospective enrolment of patients

This is a single-center retrospective observational study. We gathered information on AIS patients who had rt-PA intravenous thrombolysis at the Affiliated Hospital of Xuzhou Medical University from December 2020 to July 2022. Inclusion standards: (1) Acute ischemic stroke confirmed by CT or MRI; (2) Receive standard rt-PA intravenous thrombolytic doses within 4.5 h of AIS diagnosis (0.9 mg/Kg, the maximum dose does not exceed 90 mg) and standard usage (Within 1 min, 10% of the dose is injected intravenously, and within 60 min, the remaining 90% of the dose is pumped continuously by the intravenous route). (3) With complete data and the patients sign the informed consent. Exclusion criteria: (1) bridging artery thrombolysis or endovascular interventional therapy; (2) complicated with acute myocardial infarction, myocardial or valvular disease, myocarditis complications; (3) with a background of chronic renal failure and chronic cardiac failure; (4) A malignant tumor, an endocrine disorder, or a severe autoimmune condition; (5) The patient cannot cooperate with follow-up or other clinical data are incomplete. Informed consent was obtained from all participating patients or their legally authorized representatives. The study was approved by The Ethics Committee of the Affiliated Hospital of Xuzhou Medical University and conducted according to the Declaration of Helsinki. The enrolment flow chart is shown in Fig. 1.

### Baseline data collection

Age, gender, BMI, blood pressure (systolic and diastolic) at admission, smoking, and alcohol history, time from onset to intravenous thrombolysis(OTT), medical history (hypertension, diabetes, atrial fibrillation, coronary heart disease, history of stroke or transient ischemic attack), baseline National Institutes of Health Stroke Scale (NIHSS) score, infarct distribution (anterior circulation, posterior circulation, anterior and posterior circulation distribution), symptomatic intracranial hemorrhage (sICH) and other demographic and medical data are all included. Smoking is defined as consuming one or more cigarettes daily for a while longer than a year. Alcohol use is defined as the consumption of 100 g or more of alcohol per day for at least a year [11]. Stroke etiology was classified into four categories by the Trial of Org 10,172 in Acute Stroke Treatment (TOAST) classification [12]. Laboratory data, including NT-proBNP, low-density lipoprotein cholesterol, triglycerides, etc.

### Outcome evaluation

According to the European Cooperative Acute Stroke Study (ECASS)-III criteria [13], sICH is defined as intracranial bleeding that results in an increase in NIHSS score of ≥ 4 points after thrombolysis or death. END was characterized as an NIHSS total score rise of ≥ 4 points or the occurrence of new neurological impairments within 24 h following thrombolysis [14]. Depending on whether END happened during hospitalization following intravenous thrombolysis, patients were separated into END and non-END groups. An expert stroke center neurologist kept track of patients three months after discharge through outpatient visits or phone calls to patients and loved ones. A score of ≥ 3 on the modified Rankin Scale (mRS) indicated a bad prognosis, whereas a score of ≤ 2 indicated a good prognosis.

### Statistical analysis

The data were analyzed using SPSS 25.0 statistical software. The Shapiro–Wilk test was used to determine whether the measurement data were normal. Measurement data that fit the normal distribution were reported as mean ± standard deviation, and the groups were compared using two independent sample *t*-tests. While measurement data that did not fit the normal distribution were represented by the median (quartiles), and the non-parametric Mann–Whitney U test was employed to compare groups; frequency and percentage (%) were used to express count data, and Pearson *χ*^2^ or Fisher's exact test was employed to compare groups. On NT-proBNP, a natural log transformation [ln(NT-proBNP)] was carried out. Considering the effect of age on patients with AIS, we included age and variables with a *P* value < 0.05 in univariate analysis in a multifactorial logistic regression model to investigate potential risk factors for END and poor prognosis. Besides, we drew the ROC curve to explore the value of ln(NT-proBNP) and related factors in predicting END and poor prognosis, and the good cut-off value was calculated. *P* < 0.05 was used to denote a statistically significant difference.

## Results

### Primary results

**Fig.2:**
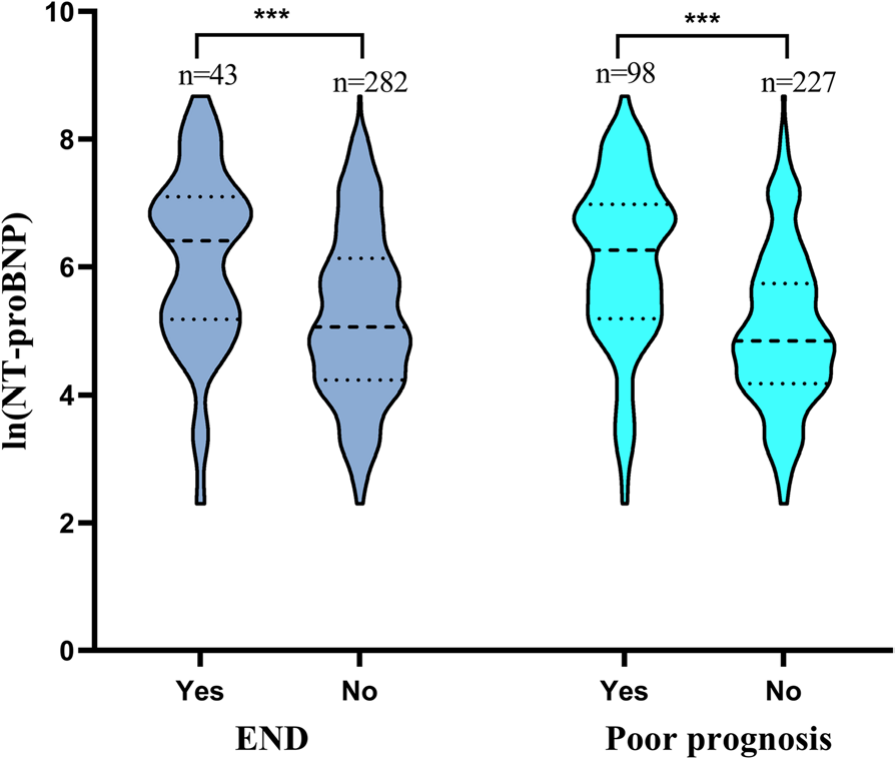
The distribution of ln(NT-proBNP) in patients with END and poor prognosis. ***P < 0.001

This research comprised 325 AIS patients, with a median age of 68 years (59–76 years) and 208 men (64%), and 117 females (36%). Preliminary findings following intravenous thrombolytic therapy showed that some patients experienced END and poor prognosis. NT-proBNP levels were higher in patients with END and poor prognosis compared to regular patients. The distribution of NT-proBNP was depicted with natural log transformations, which were depicted with median and interquartile range.The distribution of ln (NT-proBNP) levels in patients with END and poor prognosis is shown in Fig. 2. Based on the incidence of END, we divided the patients into the END group (*n* = 43) and the Non-END group (*n* = 282). Compared to patients without END, the NIHSS score, NT-proBNP, and sICH ratios in END patients were considerably higher, and the changes in infarct distribution and fasting blood glucose (FBG) were statistically significant (*P* < 0.05). As indicated in Table 1, other indicators did not alter significantly (*P* > 0.05) during the same period.

**Table 1:** Baseline data comparison between the END group and the non-END group

variable	END (*n* = 43)	Non-END (*n* = 282)	*t/x*^*2*^*/Z* value	*P* value
Male n(%)	25(58.1)	183(64.9)	0.739	0.390
Age (y)	67.00(62.00, 73.00)	68.00(59.00, 77.00)	-0.804	0.421
BMI (Kg/m^2^)	24.80(21.80, 27.30)	24.94(22.90, 27.20)	-0.683	0.495
OTT (min)	174.01(158.00, 216.00)	174.01(134.25, 207.50)	-1.471	0.141
NIHSS (score)	11.00(6.00, 16.00)	6.00(4.00, 11.00)	-3.627	< 0.001
Smoking history n(%)	19(44.2)	124(44.0)	0.001	0.979
Drinking history n(%)	18(41.9)	130(46.1)	0.270	0.603
History of atrial fibrillation n(%)	9(20.9)	51(18.1)	0.201	0.654
History of coronary heart disease n(%)	5(11.6)	55(19.5)	1.537	0.215
History of hypertension n(%)	32(74.4)	198(70.2)	0.319	0.572
History of diabetes n(%)	11(25.6)	83(29.4)	0.269	0.604
History of previous stroke or TIA n(%)	15(34.9)	84(29.8)	0.458	0.499
Symptomatic intracranial hemorrhage n(%)	13(30.2)	14(5.0)	28.043	< 0.001
Systolic blood pressure (mmHg)	160.00(139.00, 174.00)	154.00(140.00, 169.00)	-0.847	0.397
Diastolic blood pressure (mmHg)	89.00(76.00, 101.00)	86.50(76.00, 96.00)	-0.689	0.491
FBG[mmol/L, M(*P*_25_,*P*_75_)]	6.50(5.24, 9.27)	5.71(4.90, 6.57)	-2.288	0.022
Homocysteine (umol/L)	17.02(13.11, 17.02)	15.19(11.59, 18.03)	-0.807	0.420
Total cholesterol (mmol/L)	4.40 ± 1.01	4.38 ± 0.91	-0.114	0.909
Triglycerides (mmol/L)	1.09(0.82, 1.55)	1.32(0.91, 1.67)	-1.151	0.250
Low-density lipoprotein cholesterol (mmol/L)	2.77 ± 0.83	2.69 ± 0.76	-0.615	0.539
High-density lipoprotein cholesterol (mmol/L)	1.10(1.00, 1.31)	1.06(0.92, 1.20)	-1.622	0.105
Hypersensitive C-reactive protein (mg/L)	1.90(0.50, 9.90)	2.10(0.60, 5.60)	-0.163	0.870
NT-proBNP (mmol/L)	607.00(178.00, 1214.00)	157.00(68.60, 459.98)	-3.999	< 0.001
TOAST subtypes n(%)			3.209	0.357
LAA	22(51.2)	110(39.0)		
CE	6(14.0)	32(11.3)		
SAO	13(30.2)	117(41.5)		
Other	2(4.6)	23(8.2)		
Infarct distribution n(%)			8.431	0.015
Anterior circulation	10(23.3)	112(39.7)		
Posterior circulation	13(30.2)	97(34.4)		
Anterior and posterior circulation	20(46.5)	73(25.9)		

Data presented as n(%), mean ± standard deviation, or median (quartiles)

*Abbreviations*: *END* early neurological deterioration, *OTT* Onset To Treatment, *FBG* Fasting blood glucose, *NIHSS* National Institutes of Health Stroke Scale, *TIA* transient ischemic attack, *TOAST* Trial of Org 10,172 in Acute Stroke Treatment, *LAA* large-artery atherosclerosis, *SAO*, small-artery occlusion, *CE* cardioembolism

Furthermore, multivariable binary Logistic regression analysis included age and variables with a *P* < 0.05 in univariate analysis. As is shown in Table 2, the results after adjusting for the distribution of age, FBG, and infarction showed that the baseline NIHSS score (*OR* = 1.059, 95%*CI*:1.005 ~ 1.117, *P* = 0.033) and ln(NT-proBNP) (*OR* = 1.450, 95%*CI*: 1.072 ~ 1.963, *P* = 0.016) were the independent influencing factors of END after intravenous thrombolysis in AIS patient.

**Table 2:** Multivariate Logistic regression analysis of END

variable	*B* value	*B* value standard error	*Wald x*^2^ value	*P* value	*OR*	95%*CI*
NIHSS	0.057	0.027	4.524	0.033	1.059	1.005 ~ 1.117
Age	-0.027	0.017	2.593	0.107	0.974	0.943 ~ 1.006
sICH	0.914	0.533	2.948	0.086	2.495	0.879 ~ 7.086
FBG	0.064	0.061	1.080	0.299	1.066	0.945 ~ 1.202
ln(NT-proBNP)	0.372	0.154	5.799	0.016	1.450	1.072 ~ 1.963
Infarct distribution						
Anterior circulation			reference			
Posterior circulation	0.521	0.477	1.193	0.275	1.683	0.661 ~ 4.286
Anterior and posterior circulation	0.705	0.455	2.399	0.121	2.023	0.829 ~ 4.935

*Abbreviations*: *END* early neurological deterioration, *sICH* Symptomatic intracranial hemorrhage, *FBG* Fasting blood glucose, *NIHSS* National Institutes of Health Stroke Scale, *OR* Odds ratio, *CI Confidence interval*

### Secondary results

The three months of patient follow-up data were used to determine the poor and good post-AIS prognosis. Based on the results, the data was divided into poor (*n* = 98) and good prognosis (*n* = 227). Age, baseline NIHSS score, NT-proBNP, FBG, and baseline systolic blood pressure were all greater in the group with a poor prognosis than in the group with a good prognosis, as did the proportions of sICH and history of hypertension. The TOAST categorization varied between the two groups, and all variations were statistically significant (*P* < 0.05). There were no additional indicators with a significant difference (*P* > 0.05), as demonstrated in Table 3.

**Table 3:** Baseline data comparison between the good prognosis group and the poor prognosis group

variable	Good prognosis (*n* = 227)	Poor prognosis (*n* = 98)	t/x^2^/Z value	*P* value
Male n(%)	146(64.3)	62(63.3)	0.033	0.856
Age (y)	67(59.00, 75.00)	70(62.00, 80.00)	-2.134	0.033
BMI (Kg/m^2^)	25.00(22.90, 27.30)	24.47(21.73, 26.93)	-1.508	0.132
OTT (min)	174.01(143.00, 209.00)	174.01(140.00, 215.00)	-0.929	0.353
NIHSS (score)	5.00(4.00, 8.00)	11.00(7.00, 16.00)	-7.420	< 0.001
Smoking history n(%)	104(45.8)	39(39.8)	1.006	0.316
Drinking history n(%)	110(48.5)	38(38.8)	2.588	0.108
History of atrial fibrillation n(%)	39(17.2)	21(21.4)	0.821	0.365
History of coronary heart disease n(%)	45(19.8)	15(15.3)	0.928	0.335
History of hypertension n(%)	153(67.4)	77(78.6)	4.129	0.042
History of diabetes n(%)	60(26.4)	34(34.7)	2.273	0.132
History of previous stroke or TIA n(%)	62(27.3)	37(37.8)	3.524	0.061
Symptomatic intracranial hemorrhage n(%)	5(2.2)	22(22.4)	36.834	< 0.001
Systolic blood pressure (mmHg)	151.00(138.00, 168.00)	160.00(145.25, 174.25)	-3.189	0.001
Diastolic blood pressure (mmHg)	87.00(76.00, 97.00)	87.00(76.00, 96.25)	-0.313	0.754
FBG (mmol/L)	5.51(4.82, 6.52)	6.52(5.29, 9.12)	-4.493	< 0.001
Homocysteine (umol/L)	15.47(11.59, 17.50)	16.12(12.11, 17.94)	-0.104	0.917
Total cholesterol (mmol/L)	4.39 ± 0.93	4.36 ± 0.89	0.305	0.761
Triglycerides (mmol/L)	1.33(0.91, 1.72)	1.18(0.85, 1.45)	-1.695	0.090
Low-density lipoprotein cholesterol (mmol/L)	2.72 ± 0.77	2.68 ± 0.76	0.448	0.655
High-density lipoprotein cholesterol (mmol/L)	1.05(0.91, 1.20)	1.10(0.96, 1.23)	-1.601	0.109
Hypersensitive C-reactive protein (mg/L)	2.10(0.50, 5.50)	1.80(0.60, 6.43)	-0.429	0.668
NT-proBNP (mmol/L)	127.00(65.00, 312.00)	523.50(179.38, 1075.25)	-6.716	< 0.001
TOAST subtypes n(%)			8.818	0.032
LAA	84(37.0)	48(49.0)		
CE	23(10.1)	15(15.3)		
SAO	99(43.6)	31(31.6)		
Other	21(9.3)	4(4.1)		
Infarct distribution n(%)			1.433	0.488
Anterior circulation	90(39.6)	32(32.7)		
Posterior circulation	74(32.6)	36(36.7)		
Anterior and posterior circulation	63(27.8)	30(30.6)		

Data presented as n(%), mean ± standard deviation, or median (quartiles)

*Abbreviations*: *OTT* Onset To Treatment, *FBG* Fasting blood glucose, *NIHSS* National Institutes of Health Stroke Scale, *TIA* transient ischemic attack, *TOAST* Trial of Org 10,172 in Acute Stroke Treatment, *LAA* large-artery atherosclerosis, *SAO*, small-artery occlusion, *CE* cardioembolism

Furthermore, multivariate binary logistic regression analysis (MBLR) was conducted for the significant variables (*P* < 0.05) in the univariate regression analysis. According to the results of the MBLR analysis, the baseline NIHSS score (*OR* = 1.113, 95%*CI*: 1.059 ~ 1.169, *P* < 0.001), sICH (*OR* = 4.062, 95%*CI*: 1.182 ~ 13.957, *P* = 0.026), ln(NT-proBNP) (*OR* = 1.767, 95%*CI*: 1.347 ~ 2.317, *P* < 0.001) and FBG (*OR* = 1.141, 95%*CI*: 1.021 ~ 1.275, *P* = 0.020) were all independently associated with poor prognosis at three months, as demonstrated in Table 4.

**Table 4:** MBLR analysis of poor prognosis

variable	*B* value	*B* value standard error	*Wald* x^2^ value	*P* value	*OR*	95%*CI*
NIHSS	0.107	0.025	18.092	< 0.001	1.113	1.059 ~ 1.169
Age	-0.001	0.015	0.003	0.959	0.999	0.970 ~ 1.029
History of hypertension	0.585	0.368	2.530	0.112	1.795	0.873 ~ 3.690
sICH	1.402	0.630	4.954	0.026	4.062	1.182 ~ 13.957
Systolic blood pressure	0.008	0.007	1.555	0.212	1.009	0.995 ~ 1.022
FBG	0.132	0.057	5.446	0.020	1.141	1.021 ~ 1.275
ln(NT-proBNP)	0.569	0.138	16.911	< 0.001	1.767	1.347 ~ 2.317
TOAST subtypes						
LAA			reference			
CE	-0.817	0.509	2.582	0.108	0.442	0.163 ~ 1.197
SAO	0.005	0.332	0.000	0.987	1.005	0.525 ~ 1.925
Other	-0.769	0.639	1.449	0.229	0.463	0.132 ~ 1.621

*Abbreviations*: *sICH* Symptomatic intracranial hemorrhage, *FBG* Fasting blood glucose, *NIHSS* National Institutes of Health Stroke Scale, *TOAST* Trial of Org 10,172 in Acute Stroke Treatment, *LAA* large-artery atherosclerosis, *SAO*, small-artery occlusion, *CE* cardioembolism, *OR* Odds ratio, *CI Confidence interval*

**Fig. 3:**
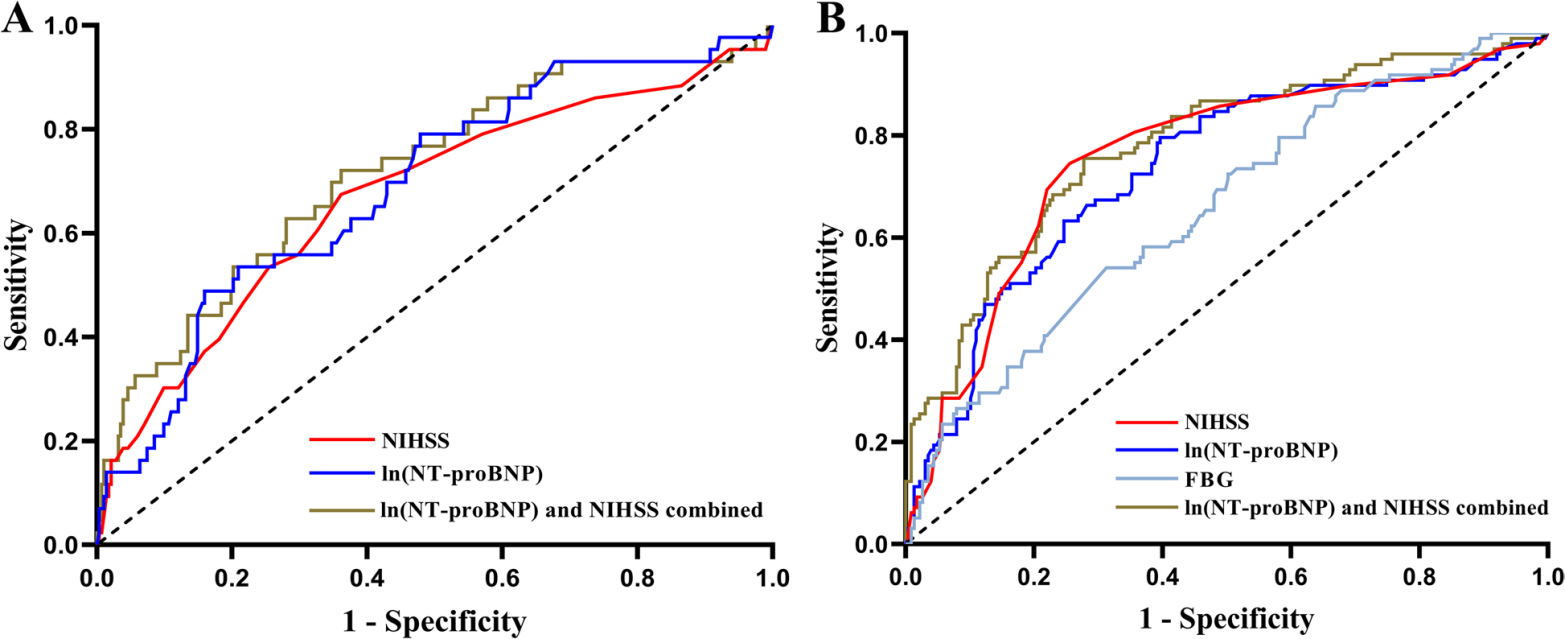
ROC curve of predictors of END and poor prognosis in AIS patients

In addition, the data was used to perform the receiving operating characteristic (ROC) to identify the predictive values of NT-proBNP along with NHISS and FBG. According to ROC curve analysis, the area under the curve (AUC) of ln(NT-proBNP) for END was 0.689 (95% *CI*: 0.604 ~ 0.775, *P* < 0.001), the cut-off value (6.64), the sensitivity (48.84%), and the specificity (84.04%). The AUC of NIHSS for predicting END was 0.671 (95%*CI*: 0.577 ~ 0.765,* P* < 0.001), the cut-off value (7.00), the sensitivity (67.44%), and the specificity (63.83%). When the two were combined (AUC 0.718, 95%*CI*: 0.631 ~ 0.805,* P* < 0.001) for predicting END, the cut-off value was 0.12, the sensitivity was 72.09%, and the specificity was 63.83%. On the other hand, the cut-off value (7.00), the sensitivity (74.49%), the specificity (74.45%), and the AUC NIHSS was 0.758 (95%*CI*: 0.698 ~ 0.818, *P* < 0.001) for the poor prognosis. FBG had an AUC of 0.657 (95%*CI*: 0.593 ~ 0.721,* P* < 0.001), the optimum cut-off value was 6.51, sensitivity was 54.08%, and specificity was 68.72%. Furthermore, with a cut-off value of 5.12, a sensitivity of 79.59%, and a specificity of 60.35%, the AUC for ln(NT-proBNP) to predict poor prognosis was 0.735 (95%*CI*: 0.674 ~ 0.796,* P* < 0.001) and when combined with NIHSS (AUC 0.780, 95%*CI*: 0.724 ~ 0.836, *P* < 0.001), the cut-off value was 0.27, with a sensitivity of 75.51% and specificity of 72.25%. Thus, NT-proBNP, like the NIHSS score, is a reliable predictor of adverse outcomes after intravenous thrombolysis in patients with AIS. When combined with the NIHSS, the model's predictive value can be further improved (Fig. 3).

## Discussion

END is a common and prominent clinical problem in AIS patients. Patients with clinical symptoms do not respond to treatment causing their families social and economic burden [5, 14, 15]. Some scholars [16] defined the END as that minor ischemic stroke patients with NIHSS scores ≤ 3 points had a slight change in NIHSS score, such as an increase of ≥ 2 points within 24 h after thrombolysis. However, there is no unified definition of END. Most studies define END as an NIHSS score rise of ≥ 4 points or death within 24 h of receiving rt-PA. It is more reasonable to adopt the minimum NIHSS score rise of ≥ 4 points as the criterion of END because the median NIHSS score of patients with AIS included in this study was 6 points. A meta-analysis [17] that included 11 independent trials used the definition to determine the END is an increase of ≥ 4 points in the NIHSS score from admission to 24 h. Additionally, they reported that the overall incidence of END following thrombolytic treatment was around 11.0% and varied from 3.4% to 28.8%. However, this study showed a strong relationship between the NIHSS score at admission and END (13.2%) occurrence and poor prognosis.

Previous studies [7, 8, 18] have demonstrated a strong correlation between END and poor neurological outcomes in AIS patients. In addition, studies [15, 18, 19] have shown that several variables and mechanisms affect END incidence, resulting in poor prognosis and disability or higher mortality in AIS patients. Common etiologies include sICH, malignant cerebral edema, early recurrent stroke, a collateral circulation disorder, and thrombus enlargement at the primary site. Furthermore, Seners et al. [16] confirmed that malignant cerebral edema and sICH are the leading direct causes of END and poor prognosis. In this study, sICH was an independent factor for END and poor prognosis at 3 months. In addition, our study found that sICH accounted for 30.2% of the END group, similar to the 20% ~ 30% of END reported by other studies [20].

**Fig. 4:**
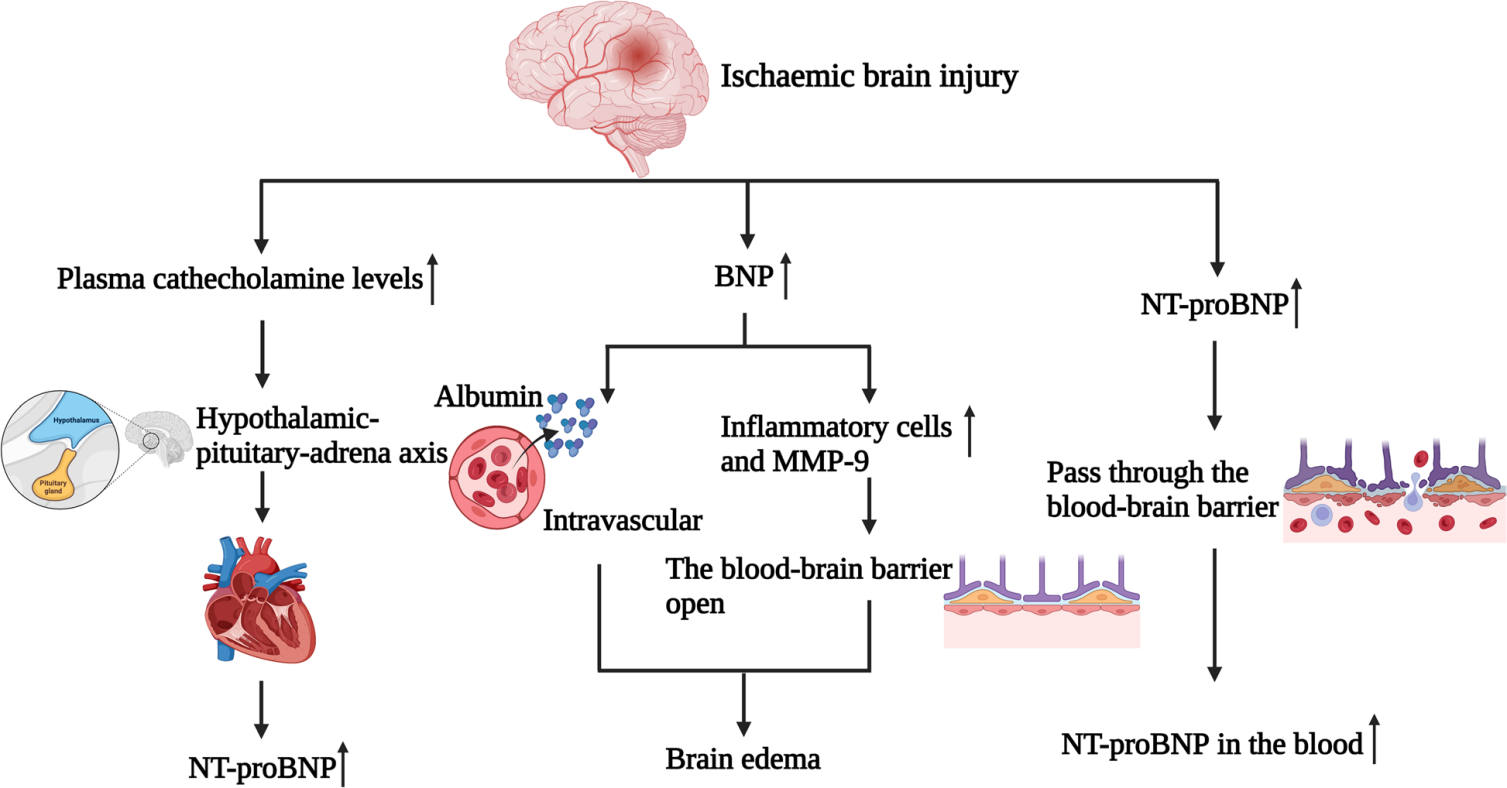
These aspects may suggest that NT-proBNP is a biomarker for predicting END and poor prognosis

The present study demonstrated that elevated blood NT-proBNP were independently associated with END and poor three-month prognosis in AIS patients. The following aspects may suggest that using NT-proBNP level as a biomarker for predicting END and poor prognosis in AIS patients receiving intravenous thrombolysis is feasible (Fig. 4). Accumulating clinical and experimental evidence [21, 22] shows that brain injury affects the heart and vice versa. The catecholamine surge hypothesis is probably the most widely accepted mechanism of brain–heart interaction. Studies [21, 23] have further demonstrated that catecholamine levels increase in response to ischemic stroke or ischemia and rt-PA-induced cerebral ischemia–reperfusion injury. High plasma catecholamine levels can lead to cardiac dysfunction by acting on cardiac adrenergic receptors to induce hypoxia and cardiomyocyte apoptosis. Ventricular dysfunction causes impaired contractility, which causes volume overload in the ventricles, stretches the ventricular myocytes, and raises NT-proBNP levels. AIS patients with ventricular systolic dysfunction may experience worsening cerebral ischemia and a higher risk of death. In addition, earlier studies [23] have demonstrated a link between neurological abnormalities and impaired cardiac function in AIS patients. Therefore, it is reasonable to assume that NT-proBNP is correlated with END and functional outcomes. Furthermore, Zhang et al. [22] demonstrated an association between high NT-proBNP levels and malignant brain edema and mortality in AIS patients after reperfusion therapy. It has been reported that BNP may play a key role in cerebral edema and cardiac function. Elevated BNP increases the permeability of microvascular endothelial cells, shifting albumin from the intravascular to the interstitial space and reducing reabsorption in extravascular areas, leading to brain edema [24]. In addition, BNP promotes the aggregation of inflammatory cells and increases matrix metalloproteinase 9(MMP-9) activity. This increase is usually associated with opening the blood–brain barrier and may lead to accelerated edema [25]. Moreover, studies [23, 26] have shown that NT-proBNP is released in the brain after brain injury and may pass through the blood–brain barrier, leading to an increase in the level of NT-proBNP in the blood, reflecting brain injury. However, its mechanism is still unclear. Moreover, Kim et al. [27] demonstrated that BNP levels in AIS patients were positively correlated with infarct volume. In addition, cardioembolic stroke and atrial fibrillation can both be predicted by high NT-proBNP levels, according to earlier researches [28, 29]. However, the findings of this study demonstrated that atrial fibrillation and cardioembolism were not independent risk factors for END and poor 3-month outcomes, which is consistent with the observations of Visnja et al. [30].

In addition, previous literature reports that [5, 14, 20] advanced age, NIHSS score, large infarction, hypertension, hyperglycemia, time from onset to thrombolysis, hs-CRP, TOAST classification, etc., closely related to poor prognosis. Further, our study showed that infarction distribution in anterior and posterior circulation significantly correlated with END, suggesting that large infarct size influenced END. The related reason may be that the formation of leptomeningeal collateral circulation in patients with higher NIHSS scores is worse, which is connected to the larger infarct volume, reflecting the severity of stroke [31]. According to a recent study, hyperglycemia on admission was strongly related to a poor prognosis in AIS patients and the results were consistent with our study [32]. Previous studies [6] have reported that various risk factors, such as advanced age and large-artery atherosclerotic stroke, lead to poor outcomes in AIS patients after rt-PA. However, it was statistically non-significant in this study. This might be related to single-center studies, small sample sizes, different designs, and sample heterogeneity. Second, we did not measure NT-proBNP levels continuously. Therefore, we could not observe a relationship between dynamic change in NT-proBNP level and functional outcomes. Future, large-sample, and multicenter clinical studies are needed to verify and generalize.

## Conclusions

In summary, this study found that NT-proBNP is an independent risk factor for END and poor prognosis in AIS patients following rt-PA and has a particular predictive value for poor prognosis. The measurement of NT-proBNP may help clinicians improve the early identification of high-risk AIS groups, adjust the treatment plan in time, and improve the clinical prognosis.

## Data Availability

Further clinical data are available from the corresponding author upon reasonable request.

## Abbreviations

END: Early neurological deterioration
FBG: Fasting blood glucose
sICH: Symptomatic intracranial hemorrhage
NIHSS: National Institutes of Health Stroke Scale
TIA: Transient ischemic attack
TOAST: Trial of Org 10,172 in Acute Stroke Treatment
LAA: Large-artery atherosclerosis
SAO: Small-artery occlusion
CE: cardioembolism
